# A retrospective study of NENs and miR-224 promotes apoptosis of BON-1 cells by targeting PCSK9 inhibition

**DOI:** 10.18632/oncotarget.14322

**Published:** 2016-12-28

**Authors:** Jian’an Bai, He Na, Xiumei Hua, Yaling Wei, Tian Ye, Yiqiang Zhang, Guo Jian, Weiwen Zeng, Lijun Yan, Qiyun Tang

**Affiliations:** ^1^ Department of Gastroenterology, Sir Run Run Hospital Affiliated with Nanjing Medical University, Nanjing, China; ^2^ Department of Gastroenterology, The First Affiliated Hospital with Nanjing Medical University, Nanjing, China; ^3^ Department of Radiology, Linyi People's Hospital, Dezhou, China; ^4^ Department of Orthopaedics, Linyi People's Hospital, Dezhou, China

**Keywords:** NENs, epidemiology, LDL-C, PCSK9, miR-224

## Abstract

Neuroendocrine neoplasms (NENs) represent relatively rare tumors. The lack of diagnostic, therapeutic method and prognostic factors makes them a challenge to us. We retrospectively reviewed the data of 205 NENs patients among which 157 cases were followed-up. Proprotein convertase subtilisin/kexin 9 (PCSK9), a regulator of low density lipoprotein cholesterol (LDL-C), was confirmed as a target gene of microRNA-224. We found an increased incidence of NENs from 2012 to 2015. Women were usually diagnosed at earlier stages than men (*P* < 0.05). Tumor grading was associated with primary tumor site, especially esophagus and cardia NENs all at G3 (*P* <0.001). Age, tumor grading and LDL-C levels were independent risk factors of digestive NENs. Low LDL-C level was significantly correlated with survival rate and median overall survival (OS, *P* < 0.05). MicroRNA-224 agomir and PCSK9 siRNA could promote apoptosis and suppress proliferation, invasion of BON-1 cells (*P* < 0.05), but increase the level of glucocorticoid (GC, *P* < 0.05). Taken together, age, tumor grading and LDL-C level are independent risk factors of NENs. The miR-224/PCSK9/GC axis binds to tumorigenesis and prognosis of pancreatic NENs (p-NENs).

## INTRODUCTION

Neuroendocrine neoplasms (NENs), ever called carcinoid, represent a highly heterogeneous and intractable disease with multiple clinical manifestations [1]. In the latest 30 years, the incidence of this disease is obviously increased with a faster growing speed than other tumors [2]. The overall incidence of digestive NENs has increased by 720% from 1972 to 2004 [3]. Most NENs are bioactive nonfunctional tumors, which lead to an early diagnostic challenge.

The acknowledged primary tumor site, grade and metastasis have been demonstrated as prognostic factors of NENs which are related to the 5-year survival rate [4, 5]. However, there is almost no other prognostic factor. As a recently discovered regulator of low density lipoprotein cholesterol (LDL-C), proprotein convertase subtilisin/kexin 9 (PCSK9) has been reported in early onset of heart disease family. It directly binds with LDL-C receptor (LDLR) on stem cells, resulting in lysosomal degradation of LDLR complex in liver and following accumulation of LDL-C in serum [6, 7]. Nevertheless, there is no report towards PCSK9/LDL-C and tumorigenesis.

MicroRNAs (miRNAs) are endogenously expressed non-coding RNAs which can bind to 3-untranslated region (UTR) of mRNAs [8]. Previous studies indicated the crucial role of miR-224 in solid tumors involving colorectal cancer, prostate cancer and hepatocellular carcinoma [9–11]. But there is no study on the effect of miR-224 in NENs.

The current paper retrospectively analyzed 205 cases with NENs and discovered LDL-C level as an independent risk factor of pancreatic NENs (p-NENs). Then, we decoded its mechanisms via miR-224/PCSK9/GC axis mediated cellular apoptosis, proliferation and invasion in p-NENs for the first time. Moreover, we demonstrated PCSK9 as a direct target of miR-224 and increased miR-224 or decreased PCSK9 could promote apoptosis and suppress proliferation, invasion of BON-1 cells in p-NENs. This may help us to evaluate the prognosis of p-NENs patients and provide a new target to the treatment of p-NENs.

## RESULTS

### Epidemiology

**Figure 1:**
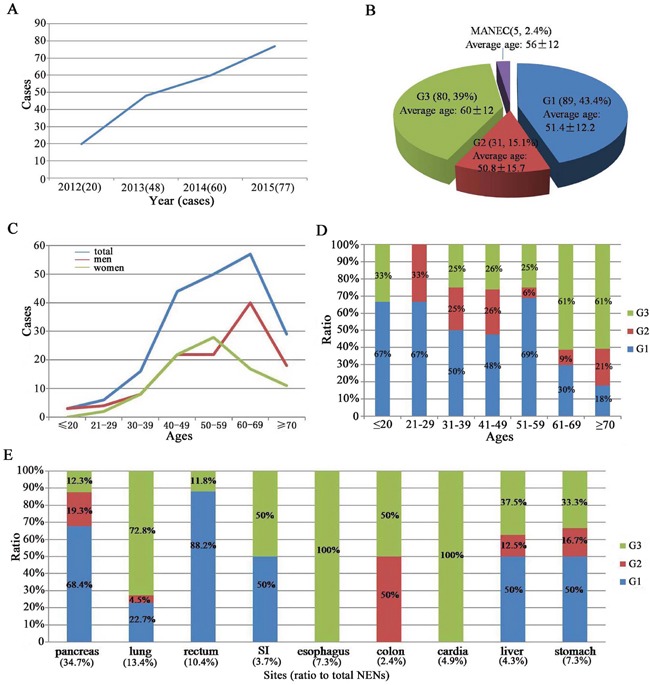
Epidemiology of 205 NENs patients **A**. a significant increase was shown in the incidence of NENs from 2012 to 2015. **B**. distribution of patients according to stage and the mean age at diagnosis. **C**. the most common age of NENs in men and women. **D**. the relationship between tumor grading and the age. **E**. the common primary tumor site and the relationship between tumor grading and the primary tumor site.

A total of 205 NENs were involved and showed a significant increase in incidence of NENs from 2012 (n = 20) to 2015 (n = 77, Figure 1A). There were 89 patients (43.4%) with NENs G1, 31 patients (15.1%) with NENs G2, 80 patients (39%) with NENs G3 and 5 patients (2.4%) with mixed adenoneuroendocrine carcinoma (MANEC). Mean age of G3 and MANEC was older than G1 and G2 with no significant difference (*P* = 0.213, Figure 1B). The most common age of NENs was 60 to 69 years in men while 50 to 59 years old in women (*P* < 0.05, Figure 1C). Tumor grading was strongly associated with age, especially > 60 years (*P* < 0.001, Figure 1D).

The most common primary tumor site was pancreas (n = 57; 34.7%), followed by lung (n = 22; 13.4%), rectum (n = 17; 10.4%), esophagus (n = 12; 7.3%), stomach (n = 12; 7.3%), cardia (n = 8; 4.9%), small intestine (SI, n = 6; 3.7%). Tumor grading was strongly associated with primary tumor site, especially esophagus and cardia all at NENs G3. However, pancreatic and rectal NENs were always at G1 (*P* <0.001, Figure 1E). Clinical characteristics of these patients were presented in Table 1. About 91.7% NENs were nonfunctioning tumors. Abdominal pain was the most common complaint (only 13.7% of 205 cases).

**Table 1:** Clinical characteristics of NENs patients

	abdominal pain	diarrhea	flush	syncope	hemoptysis
pancreas	16 (22.2%)	0	4 (5.6%)	11 (15.5%)	0
cardia	3 (30%)	0	0	0	0
SI	3 (37.5%)	0	0	0	0
colon	1 (25%)	0	0	0	0
rectum	4 (19%)	2 (9.5%)	0	0	0
stomach	1 (6.7%)	0	0	0	0
liver	0	0	0	0	0
lung	0	0	0	0	11(40.7%)

### Levels of serum lipid

The average levels of serum lipid in NENs patients were significantly lower than the healthy examinees including levels of total triglyceride (TG), total cholesterol (TC), high density lipoprotein cholesterol (HDL-C) and LDL-C (Supplementary Table 1), especially the LDL-C level (2.55 ± 0.8 *vs* 3.5 ± 0.85 mmol/L, *P* < 0.001, Supplementary Figure 1A).

There were more cases with high LDL-C (>4.1 mmol/L) in healthy examinees than those in NENs patients (42/200 *vs* 9/205, *P* < 0.001) and more cases with low LDL-C (≤2.6 mmol/L) in NENs patients than those in healthy examinees (26/200 *vs* 61/205, *P* = 0.001, Supplementary Table 2).

### Survival

**Figure 2:**
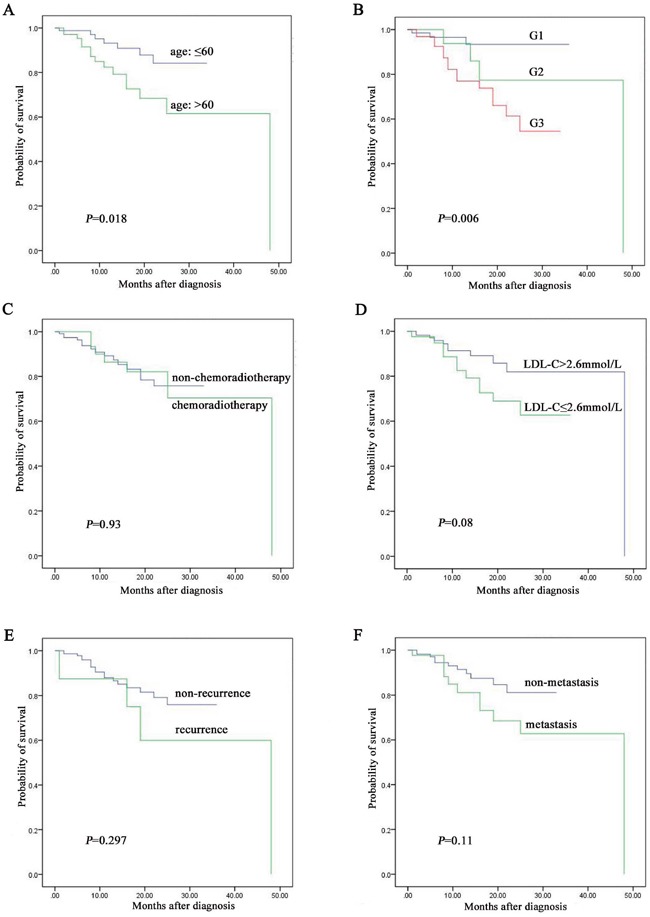
The prognostic factors assay by Kaplan-Meier analysis among the 157 NENs patients followed up **A**. age was associated with prognosis of NENs. **B**. grading was associated with prognosis of NENs. **C**. chemoradiotherapy was not associated with prognosis of NENs. **D**. level of LDL-C was not associated with prognosis of NENs. **E**. recurrence was not associated with prognosis of NENs. **F**. metastasis was not associated with prognosis of NENs.

Among the 157 cases followed up, patients less than 60 years old were associated with better prognosis than over 60 years group (*P* = 0.018, Figure 2A). Diagnosis of NENs G1 was associated with better prognosis than G2 and G3 (*P* = 0.006, Figure 2B). However, there was no significant correlation between survival and chemoradiotherapy (*P* = 0.93, Figure 2C), level of LDL-C (*P* = 0.08, Figure 2D), recurrence (*P* = 0.297, Figure 2E) and metastasis (*P* = 0.11, Figure 2F).

**Figure 3:**
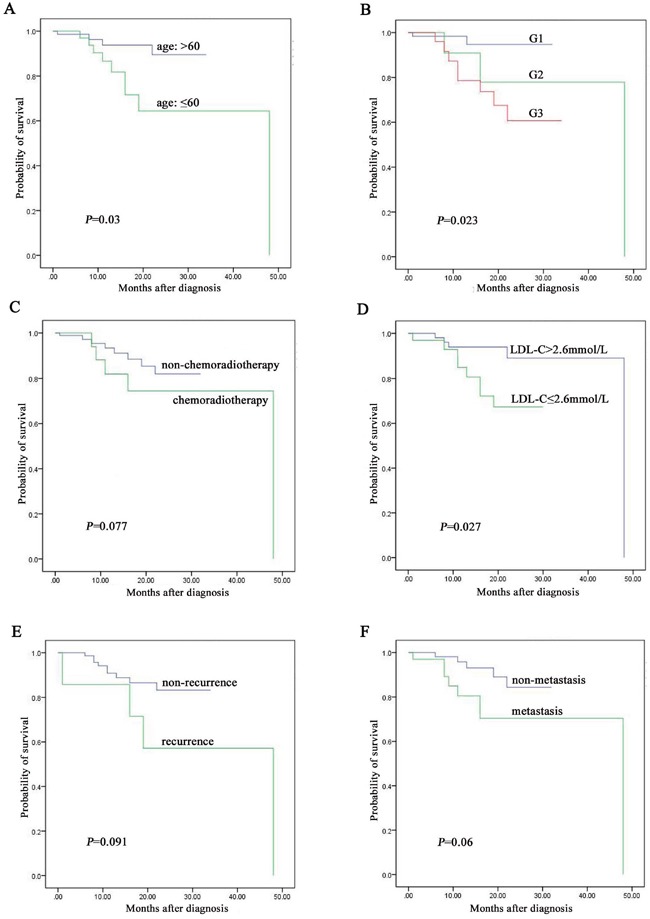
The prognostic factors assay by Kaplan-Meier analysis among the 116 digestive NENs patients followed up **A**. age was associated with prognosis of digestive NENs. **B**. grading was associated with prognosis of digestive NENs. **C**. chemoradiotherapy was not associated with prognosis of digestive NENs. **D**. level of LDL-C was associated with prognosis of digestive NENs. **E**. recurrence was not associated with prognosis of digestive NENs. **F**. metastasis was not associated with prognosis of digestive NENs.

Among the 116 cases of digestive NENs followed up, patients less than 60 years old were associated with better prognosis than over 60 years group (*P* = 0.03, Figure 3A). Diagnosis of NENs G1 was associated with better prognosis than G2 and G3 (*P* = 0.023, Figure 3B). Patients with high LDL-C level were associated with better prognosis than low LDL-C level group (*P* = 0.027, Figure 3D). However, there was no significant correlation between survival and chemoradiotherapy (P = 0.077, Figure 3C), recurrence (*P* = 0.091, Figure 3E) and metastasis (*P* = 0.06, Figure 3F). Otherwise, patients with high LDL-C level owned 4.738-fold of survival rate than low LDL-C level group (95% CI, 1.424 to 15.772, *P* = 0.019). LDL-C level was significantly correlated with survival rate (r = -0.23, *P* = 0.013) but not significantly correlated with survival time (r = -0.055, *P* = 0.557). In detail, the median overall survival (OS) in low LDL-C level group was 50.6±16.2 months, 56.9±11.7 months in normal LDL-C level group (2.6-4.1 mmol/L) and 51.1±7.6 months in high LDL-C level group (*P* = 0.039).

### PCSK9 is the direct target of miR-224

**Figure 4:**
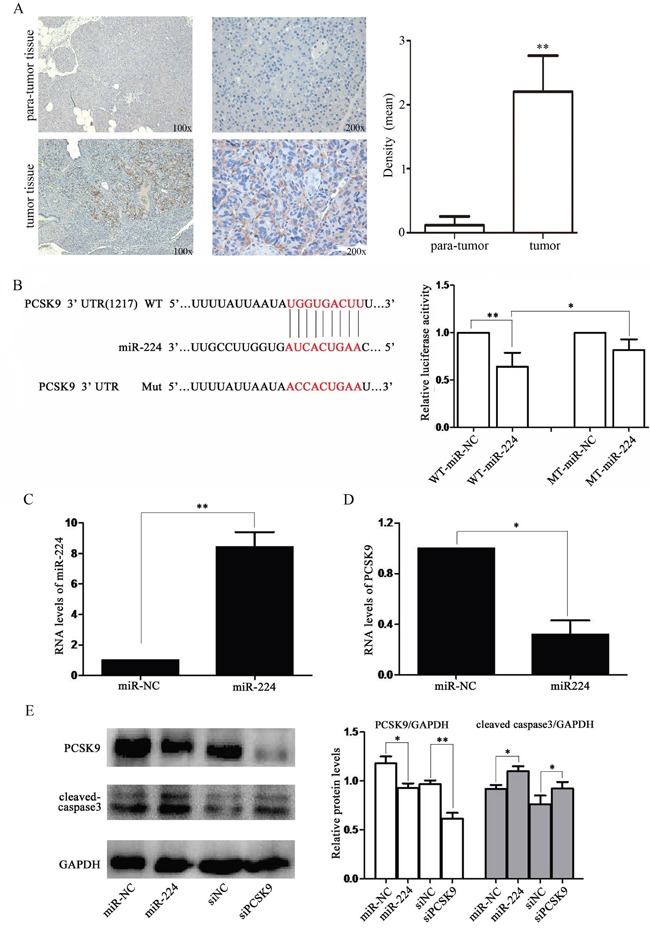
miR-224 down-regulates PCSK9 expression by directly targeting its 3′-UTR **A**. PCSK9 expression was increased in tissue samples of p-NENs. Immunohistochemistry of PCSK9 in tumor and para-tumor samples from patients with NENs (n=6, 3 males and 3 females). There was almost non-staining of PCSK9 in the para-tumor samples, while strong staining of PCSK9 in tumor samples. **B**. RNA sequence alignment showed the 3′-UTR of PCSK9 mRNA contains a complementary site for miR-224. Dual luciferase reporter assay was performed to confirm the miR-224 binding target. **C, D**. RT-PCR analysis showed that the miR-224 expression was up-regulated and PCSK9 expression was down-regulated in BON-1 cells upon miR-224 transfection at mRNA levels. **E**. western blot analysis showed that the endogenous PCSK9 expression was significantly reduced at protein level followed by increased expression of cleaved caspase-3 upon miR-224 agomir or siPCSK9 transfection.

PCSK9 was an effective regulator of LDL-C as previous reported. We detected expression of PCSK9 in tissue samples of p-NENs and found a significantly increase than para-tumor tissues (*P* < 0.01, Figure 4A). Therefore, we chose it as the following target. First, we used miRNA target predicting programs (Target-Scan) to discover PCSK9 as a target of miR-224. Then, luciferase reporter was performed to confirm PCSK9 being targeted by miR-224. PCSK9 reporter was significantly decreased upon co-transfection with miR-224 agomir, but not in control group with mutated sequence (*P* < 0.05, Figure 4B).

Upon transfection with miR-224 agomir, miR-224 level was significantly increased (*P* < 0.01, Figure 4C). Meanwhile, endogenous PCSK9 expression was significantly reduced at mRNA level (*P* < 0.05, Figure 4D) and protein level (*P* < 0.05, Figure 4E). Therefore, we believed that PCSK9 was a direct target of miR-224.

### miR-224 agomir or siPCSK9 promotes apoptosis and suppresses proliferation, invasion of BON-1 cells *in vitro*

**Figure 5:**
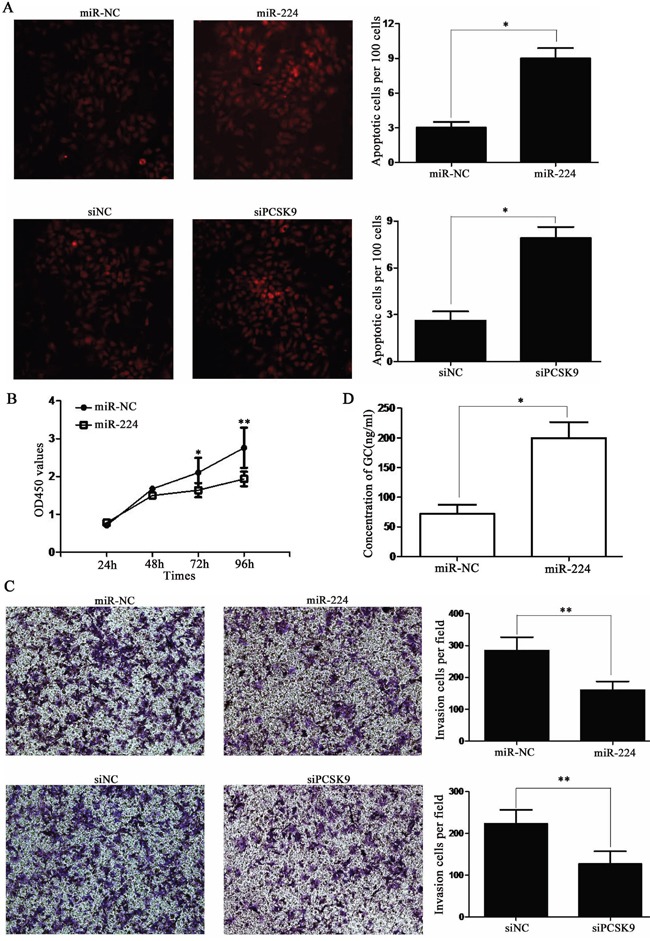
miR-224 agomir or siPCSK9 promotes apoptosis and suppresses proliferation, invasion of BON-1 cells **A**. the TUNEL-positive cells was about 3% in miR-NC group and up to 8.9% in miR-224 agomir group. Meanwhile, it was 2.7% in siNC group and up to 8.2% in siPCSK9 group. **B**. CCK8 assay showed significantly lower proliferation potential in miR-224 agomir transfected cells than control group after 72 h. **C**. transwell assay showed significantly less invasive cells in miR-224 agomir group or siPCSK9 group than in control group. **D**. ELISA assay showed an increased level of GC in miR-224 agomir group than in miR-NC group.

Then, we detected cleaved caspase-3 level which was a “apoptotic executor” in mitochondrial apoptosis [12]. A significantly increased cleaved caspase-3 level was found in miR-224 agomir or siPCSK9 transfected BON-1 cells than negative control (NC) group (*P* < 0.05, Figure 4E). The percentages of cell death or TUNEL-positive cells were about 3% in miR-NC group and up to 8.9% in miR-224 agomir group (*P* < 0.05, Figure 5A). Meanwhile, it was 2.7% in siNC group and up to 8.2% in siPCSK9 group (*P* < 0.05, Figure 5A). Furthermore, we found a significantly lower proliferation potential in miR-224 agomir transfected BON-1 cells than miR-NC group after 72 h (*P* < 0.05, Figure 5B) with CCK8 assay. The same result was seen in siNC and siPCSK9 group (*P* < 0.05, Supplementary Figure 1B).

In transwell assay, there were average of 282 invasive cells in miR-NC group and only 138 invasive cells in miR-224 agomir group. Meanwhile, there were average of 215 invasive cells in siNC group and only 127 invasive cells in siPCSK9 group (*P* < 0.05, Figure 5C).

Therefore, we performed an enzyme-linked immuno sorbent assay (ELISA) assay to measure GC level and found an increased level of GC in miR-224 agomir group than in miR-NC group (*P* < 0.05, Figure 5D) and the same result in siPCSK9 group than siNC group (*P* < 0.05, Supplementary Figure 1C).

### No tumor formation in the nude-mice with BON-1 cells

To finally confirm the function of miR-224 and PCSK9, we performed tumor formation assay *in vivo*. About 5×10^6^ BON-1 cells/mouse and 1×10^7^ BON-1 cells/mouse with or without pre-transfection were employed. Regrettably, there was no tumor formation in any mice. These results might indicate disability of BON-1 cells in tumor formation.

## DISCUSSION

Since 1907, we have paid attention to them for a long way of more than 100 years. However, that is still a big challenge about early diagnosis and effective treatment [3, 13]. Here, we retrospectively analyzed 205 cases of NENs in our hospital about clinical data and demonstrated a significant increase in incidence of NENs in recent 4 years.

Some discrepancies to previous studies or results still exist: (1) the most common primary tumor site is pancreas, followed with small intestine; (2) tumor grading was strongly associated with primary tumor site, especially esophagus and cardia all at NENs G3; (3) level of LDL-C is significantly correlated with the survival rate and OS [2–4, 14–16]. The most important thing is that esophageal and cardiac NENs need more attentions owing to the bad differentiation.

LDL-C is known as adverse cholesterol and raw material of GC synthesis which plays a critical role in cellular apoptosis [17, 18]. Several studies have showed effect of GC on lipid metabolism [19], but seldom explored effect of lipid level on GC synthesis. Ronald et al have reported for the first time an inhibitory role of LDLR in GC production [20]. However, we found a discrepant result in our paper. An increasing level of GC was shown upon up-regulation of miR-224 or down-regulation of PCSK9. We considered it as a dynamic state. In detail, LDLR-mediated degradation of LDL-C released more raw material of GC synthesis, inducing accumulation of GC in plasma.

PCSK9 is responsible for degradation of the LDL-C receptor at the cell surface, and thus has a direct effect on intracellular and serum LDL-C levels [21]. It has only been reported as a target gene of two miRNA (miR-27a and miR-124) [22, 23]. However, relationship between miR-224 and PCSK9 is still unknown. MiR-224 has been reported to correlated with apoptosis, proliferation, cell cycle and invasion of several cancers especially the lung cancer and colon cancer [24–26]. In our study, we demonstrated PCSK9 as a direct target of miR-224. Moreover, we uncovered that increased miR-224 or decreased PCSK9 could promote apoptosis and suppress proliferation, invasion of BON-1 cells in pNENs.

In conclusion, we demonstrate that age, tumor grading and LDL-C level are independent risk factors of digestive NENs. Level of LDL-C is significantly correlated with survival rate and OS of NENs patients. MiR-224/PCSK9/GC axis closely correlated with carcinogenisis and prognosis of p-NENs which may provide a new target to treatment of p-NENs.

## MATERIALS AND METHODS

### Patients and immunohistochemistry

Epidemiological study involved 205 patients (117 men, 88 women) from database of record room in our hospital from 2012 to 2015, with diagnosis of NENs according to WHO 2010 criteria [2]. A total of 157 patients including 116 cases of digestive NENs were successfully followed-up. We summarized clinical features and prognostic factors including age, sex, grading (G1, G2, G3 without MANEC), distant metastases at diagnosis, chemoradiotherapy and relapse. Date for survival analyses was from diagnosis until death. Serum lipid levels of NENs patients and 200 healthy examinees with similar age and sex were retrospectively collected at the first coming to our hospital before surgery. This study was approved by ethics committee of the First Affiliated Hospital with Nanjing Medical University.

Tissues of p-NENs patients (n=6) were gained from pathology department in our hospital. Immunohistochemistry assay was performed according to manufacturer's protocol. The immunohistochemical kit was used (Beyotime Co., Ltd, China) for all procedures. Para-tumor samples were served as negative controls. These slices were visualized with a microscope (IX71; Olympus Corp., Tokyo, Japan).

### Cell culture and transfection

Human p-NENs cell lines BON-1 was obtained from American Type Culture Collection and authenticated with short tandem repeat (STR, JiaPeng Co., Ltd, China). Cells were cultured in DMEM medium (Hyclone) added with 10% FBS (Siencell, USA) conventionally.

The miRNA-224 agomir, agomir negative control, PCSK9 siRNA (siPCSK9) and siRNA negative control (siNC) were designed (GenePharma Co.,Ltd, China). The sequence of miRNA-224 agomir was 5′-CAAGUCACUAGUGGUUCCGUU-3′ (sense), 5′-CGGA ACCACUAGUGACUUGUU-3′ (antisense). Sequence of siRNA against PCSK9 was shown as follows: 5′-CCAAGAUCCUGCAUGUCUUTT-3′ (sense), 5′-AAG ACAUGCAGGAUCUUGGTT-3′ (antisense). Sequence of siRNA negative control was as follows: 5′-UUCU CCGAACGUGUCACGUTT-3′ (sense), 5′- ACGU GACACGUUCGGAGAATT-3′ (antisense). Transfected experiments were performed with Lipofectamine 2000 (Invitrogen) according to manufacturer's procedure. BON-1 cells were respectively cultured with miRNA agomir, siRNA, or negative control for 48 hours.

### MiRNA target prediction and luciferase reporter assay

Potential targeted relation of PCSK9 and miR-224 was predicted with Target-Scan. Results were assessed with luciferase reporter assay in BON-1 cells. BON-1 cells were co-cultured with miR-224 agomir or negative control (50 nmol/L), and then wild-type (WT) or mutant (MT) reporter plasmid (0.5 mg). Cells were collected 48 hours after transfection and measured with dual-luciferase reporter assay system (Promega, USA). Data were normalized to renilla luciferase signal.

### Western blot

For immunoblot analysis, about 80 μg of routinely distracted cellular protein was resolved on a 10% sodium dodecyl sulfate polyacrylamide electrophoresis gel. PCSK9 (1:6000), cleaved-caspase 3 (1:6000) and glyceraldehyde-3-phosphate dehydrogenase (GAPDH, 1:6000) were served as primary antibodies (Abcam, USA). GAPDH was served as an internal control. The membranes were exposed to bands with a Chemiluminescent HRP Substrate (Millipore, MA, USA).

### RNA isolation and real-time PCR

Trizol RNA extraction kit, reverse transcription kit and SYBR Green PCR Kit (Takara, Japan) were used for real-timePCR assay according to the manufacture's protocol on StepOne-PCR System (Applied Biosystems). Relative expression ratio of target genes was calculated by 2^−ΔΔCT^ method. Expression levels of miR-224 or PCSK9 were normalized to U6 or GAPDH, respectively. Specific primers of PCSK9 were 5′-AGACCCACCTCTCGCAGTC-3′ (sense); 5′-GGAGTCCTCCTCGATGTAGTC-3′ (antisense);

Primers of GAPDH were 5′-CTGGGCTACA CTGAGCACC-3′ (sense); 5′-AAGTGGTCGTTGAGG GCAATG-3′ (antisense). MiR-224 and U6 were designed and synthesized (Ribobio Co., Ltd, China).

### TUNEL and CCK-8 assay

TdT-UTP nick-end labeling (TUNEL; Beyotime Co., Ltd, China) assay was performed according to manufacture's protocol. Fluorescein isothiocyanate (FITC)-labeled TUNEL-positive cells were imaged with a microscope. Cells with deep red fluorescence were defined as apoptotic cells.

A Cell Counting Kit-8 (CCK-8) assay was performed following manufacturer's protocol to evaluate cellular proliferation. Each sample was supplemented with 10 μl tetrazolium substrate after transfection for 24, 48, 72 and 96 h. Optical density (OD) was then read at 450 nm in a spectrophotometer (Bio-Tek, USA).

### ELISA assay

To evaluate levels of glucocorticoid, human Enzyme-linked immunosorbent assay (ELISA, Senbeijia Co., Ltd, China) was performed according to manufacturer's protocol. Absorbance was measured at 450 nm and levels of glucocorticoid were read out from respective standard curve.

### Transwell assay

Cell invasion viability was performed by Transwell assay according to the protocol in previous studies [27]. Migrated cells were photographed and counted at 6 random fields of each group.

### Animals and tumor formation assays

Animal experiments were supported by the Institute for Laboratory Animal Research at Nanjing Medical University. Twelve BALB/c nude mice (4 to 5 weeks old, females) were purchased from Animal Center of Jiangsu province. Pre-transfected BON-1 cells with miR-224 agomir or control agomir were collected in PBS. The highest concentration of 1×10^7^ cells were subcutaneously injected into oxter of each mouse (4/group). Situation and tumor formation were observed and recorded every 2 days.

### Statistical analysis

Pictures drawing and statistical analysis were performed with GraphPad prism 5 and SPSS (version13.0). Differences between two groups were estimated with Student's *t* test. Continuous variables were expressed as mean ± SD. The Pearson χ^2^ test or Fisher exact test was performed for crosstables. The univariate analysis was performed with Kaplan-Meier method and compared with log-rank tests for the survival rate. Pearson relevant analysis was calculated between levels of LDL-C and overall survival and OS. A *P* value of less than 0.05 was considered statistically significant.

## SUPPLEMENTARY MATERIALS FIGURE AND TABLES


