# Supplementary material for: A retrospective study of NENs and miR-224 promotes apoptosis of BON-1 cells by targeting PCSK9 inhibition
Source: Oncotarget. 2016 Dec 28;8(4):6929–39. doi: 10.18632/oncotarget.14322 (PMC5351680; doi:10.18632/oncotarget.14322)
Supplement: Supplementary file 1 [file oncotarget-08-6929-s001.pdf]

A retrospective study of NENs and miR-224 promotes apoptosis of BON-1 cells by targeting PCSK9 inhibition

SUPPLEMENTARY FIGURE AND TABLES

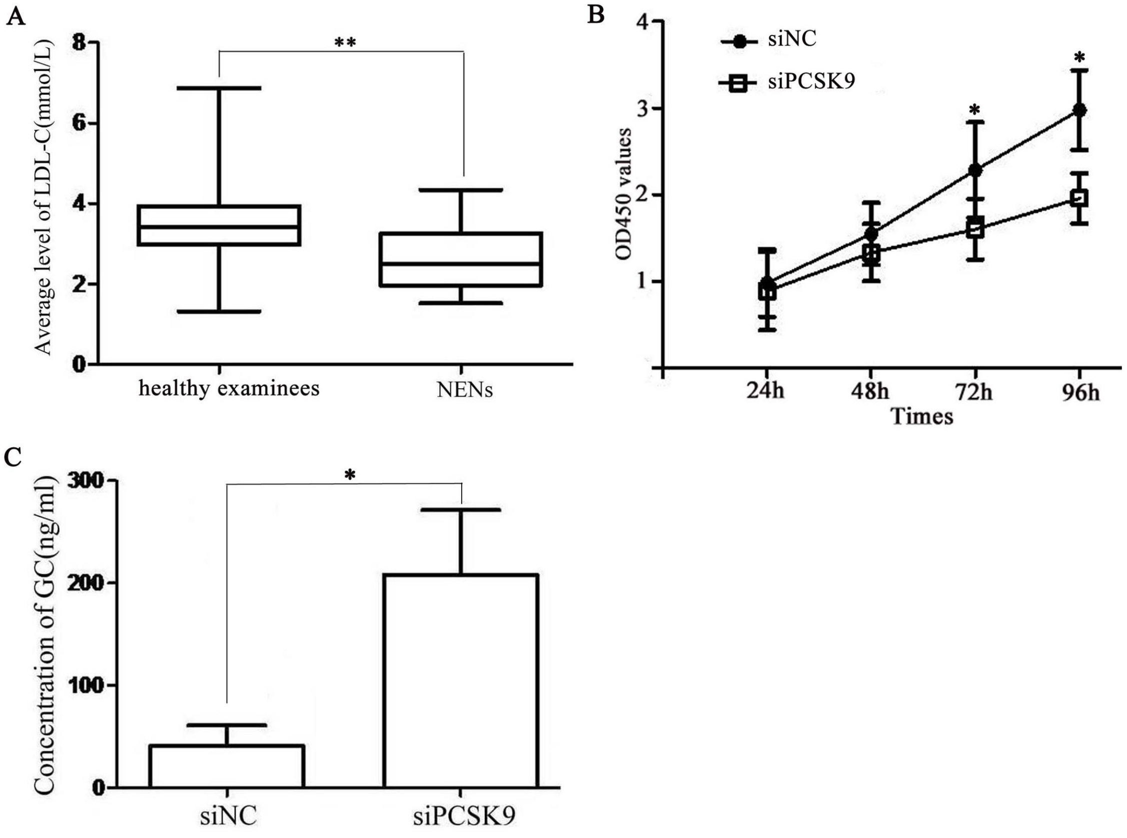

Supplementary Figure 1: A. The average level of LDL-C in NENs patients than the healthy examinees. **B.** CCK8 assay showed significantly lower proliferation potential siPCSK9 transfected cells than siNC group after 72 h. **C.** ELISA assay showed an increased level of GC in siPCSK9 group than in siNC group.

Supplementary Table 1: Average levels of plasma lipid in NENs patients and healthy examinees

	TG	TC	HDL-C	LDL-C
	Mean±SD			
healthy examinees	1.87 ± 1.26	5.30 ± 1.00	1.27 ± 0.28	3.5 ± 0.85
NENs patients	1.22 ± 0.77	4.14 ± 1.04	1.08 ± 0.28	2.55 ± 0.8
P	0.005	0.000	0.001	0.000

Supplementary Table 2: Cases with high level or low levels of LDL-C in NENs patients and healthy examinees

	High LDL	Low LDL
healthy examinees	42/200	26/200
NENs patients	9/205	61/205
P	0.000	0.001
